# The Stimulatory Effect of Strontium Ions on Phytoestrogens Content in *Glycine max* (L.) Merr

**DOI:** 10.3390/molecules21010090

**Published:** 2016-01-14

**Authors:** Magdalena Wójciak-Kosior, Ireneusz Sowa, Tomasz Blicharski, Maciej Strzemski, Sławomir Dresler, Grażyna Szymczak, Artur Wnorowski, Ryszard Kocjan, Ryszard Świeboda

**Affiliations:** 1 Department of Analytical Chemistry, Medical University of Lublin, Chodźki 4a, Lublin 20-093, Poland; i.sowa@umlub.pl (I.S.); maciej.strzemski@poczta.onet.pl (M.S.); r.kocjan@umlub.pl (R.K.); 2 Orthopaedics and Rehabilitation Clinic, Medical University Lublin, Chodźki 4a, Lublin 20-093, Poland; blicharski@vp.pl; 3 Department of Plant Physiology, Institute of Biology and Biochemistry, Maria Curie-Skłodowska University, Akademicka 19, Lublin 20-033, Poland; slawomir.dresler@poczta.umcs.lublin.pl; 4 Botanical Garden of Maria Curie-Skłodowska University in Lublin, Sławinkowska 3, Lublin 20-810, Poland; grazyna.szymczak@poczta.umcs.lublin.pl; 5 Department of Biopharmacy, Medical University of Lublin, Chodźki 4a, Lublin 20-093, Poland; artur.wnorowski@gmail.com; 6 Department of Inorganic Chemistry, Medical University of Lublin, Chodźki 4a, Lublin 20-093, Poland; r.swieboda@umlub.pl

**Keywords:** soy, *Glycine max*, phytoestrogens, isoflavones, coumestrol, strontium.

## Abstract

The amount of secondary metabolites in plants can be enhanced or reduced by various external factors. In this study, the effect of strontium ions on the production of phytoestrogens in soybeans was investigated. The plants were treated with Hoagland’s solution, modified with Sr^2+^ with concentrations ranging from 0.5 to 3.0 mM, and were grown for 14 days in hydroponic cultivation. After harvest, soybean plants were separated into roots and shoots, dried, and pulverized. The plant material was extracted with methanol and hydrolyzed. Phytoestrogens were quantified by HPLC. The significant increase in the concentration of the compounds of interest was observed for all tested concentrations of strontium ions when compared to control. Sr^2+^ at a concentration of 2 mM was the strongest elicitor, and the amount of phytoestrogens in plant increased *ca.* 2.70, 1.92, 3.77 and 2.88-fold, for daidzein, coumestrol, genistein and formononetin, respectively. Moreover, no cytotoxic effects were observed in HepG2 liver cell models after treatment with extracts from 2 mM Sr^2+^-stressed soybean plants when compared to extracts from non-stressed plants. Our results indicate that the addition of strontium ions to the culture media may be used to functionalize soybean plants with enhanced phytoestrogen content.

## 1. Introduction

Phytoestrogens are a diverse group of nonsteroidal plant metabolites, including three main classes of phenolic compounds: coumestans, prenylflavonoids and isoflavones. They are produced by many plants such as the red clover (*Trifolium pratense* L.) and the kudzu (*Pueraria lobata*, Willd.) [1]. However, soybeans (*Glycine max* L. Merr.; *Glycine hispida* Moench Maxim; *Phaseolus max* L.; *Soya max* L. Piper) in the legume family (*Fabaceae*) and soy-based products, e.g., soy milk and tofu, are the most valuable sources of phytoestrogens, as they may be easily incorporated into the human diet [2,3]. The biological activity of soy isoflavones is the best known and has been confirmed by numerous scientific studies. It has been reported that mixtures of soy isoflavones containing 14.3% genistein, 14.7% glycitein and 71% daidzein at concentration 5–80 µg/mL effectively inhibit growth of cervical cancer cell in *in vitro* assay [4]. Genistein showed significant antileukemic effects in murine leukaemia model *in vivo* [5] and displayed chemoprotective actions at a concentration of 10 µM in *in vitro* assay on hepatoma cells [6]. Isoflavones belong to derivatives of 3-phenyl-chromen-4-one and, due to the structural similarity to β-estradiol, possess estrogenic activity [7]. Moreover, they decrease the risk of cancer and have antioxidant, antibiotic, anti-inflammatory and anti-allergic properties [8,9]. They also protect against some chronic diseases related to aging, such as cardiovascular diseases and osteoporosis. According to recent research on menopausal women, soy pharmaceutical formulations containing a standardized amount of isoflavones induce bone formation, increase bone mineral density and alleviate the symptoms of osteoporosis [10,11]. Since strontium, as a strontium ranelate, at a dose of 2 g per day is also used in therapy of human osteoporosis [12], biofortification of *Glycine max* (*G. max*) with this element may be beneficial to human health. In our previous study, a relatively high ability of soybeans to accumulate strontium ions as well as absence of toxicity or of negative effects of up to 2.0 mM strontium concentrations on growth and physiological parameters were reported [13]. However, the literature indicates that stress conditions such as extensive sun exposure, water deficit, salinity and elevated levels of metal ions in the growth medium may affect the production of secondary metabolites [14,15,16]. The response of plants exposed to stress depends on the species and stress factors, and may include both a negative [17,18] and a stimulatory effect [19,20].

Therefore, in the present paper, the influence of strontium ion additions to growth mediums on the content of phytoestrogens in *G. max* was investigated. In order to assess the safety of the extracts to human cells in culture, the HepG2 liver cell model was employed.

## 2. Results and Discussion

### 2.1. HPLC Analysis

**Figure 1:**
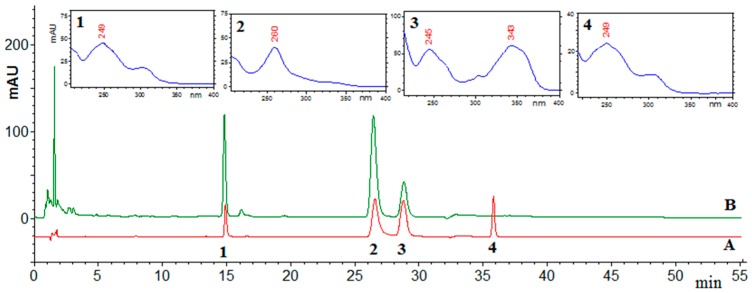
The example of chromatogram: A—mixture of standard and B—hydrolyzed strontium stressed soy extract (1—daidzein, 2—genistein, 3—coumestrol, and 4—formononetin).

Various derivatives of isoflavones (e.g., malonyl or acetyl glycosides) are found in soybeans. Most of them are not commercially available; thus, conversion of bonded forms to aglycones is often used for quantitative determination. In our research, acidic hydrolysis according to Shao *et al.* [21] was used prior to the chromatographic analysis for quantification of the total amount of isoflavones. The chromatographic conditions for analysis of the extracts were elaborated on the basis of previously reported data [22]. The gradient program was modified experimentally to achieve separation of isoflavones from the other compounds over a relatively short time (Figure 1).

The identity of compounds was established by a comparison of retention times and UV-Vis spectra with standards (the similarity factor calculated by EZChrom Elite software was higher than 0.98 for all compounds) and confirmed by direct-injection mass spectrometry ([M + H]^+^). The values were as follows: For daidzein t_R_ = 14.9, λ_max_ = 249, *m/z* = 255; for genistein t_R_ = 26.2, λ_max_ = 260, *m/z* = 271; for coumestrol t_R_ = 28.7, λ_max_ = 343, *m/z* = 269; and for formononetin t_R_ = 35.8, λ_max_ = 249, *m/z* = 269. Moreover, the MS spectra obtained were compared with data from High Quality Mass Spectral Database (MassBank).

The calibration curves for quantitative HPLC analysis were constructed on the basis of the relationship between peak areas *versus* standard concentrations at five concentration levels (*n* = 5). The accuracy of this method was established by performing recovery experiments at three different levels. The limit of detection (LOD) and limit of quantification (LOQ) were established on the basis of signal-to-noise ratio, which was 3 and 10, respectively. The validation parameters obtained are presented in Table 1. Five injections of each sample were used for quantification, and the amounts of the investigated compounds were calculated using the calibration equations. The specificity of the method was checked by an acquisition of spectra at three different peak sections (upslope, apex and downslope) and by a comparison with a reference spectrum.

**Table 1:** Validation parameters (*n* = 5).

Parameter	Daidzein	Genistein	Formononetin	Coumestrol
Concentration range (μg·mL^−1^)	1.0–50.0	0.01–30.0	0.01–5.0	0.01–10.0
Regression equation	y = 396815x + 104631	y = 398812x − 155217	y = 509672x − 245893	y = 118314x − 67144
Correlation coefficient R	0.9998	0.9999	0.9999	0.9999
Precision (* RSD %)	0.5–1.1	0.6–1.2	0.6–1.0	0.5–1.3
Accuracy (mean recovery %)	98.72	98.91	97.58	98.11
LOD (ng·mL^−1^)	18.2	25.0	28.1	27.4
LOQ (ng·mL^−1^)	60.7	83.3	93.7	91.3

* RSD—relative standard deviation.

### 2.2. The Effect of Strontium Concentration on the Phytoestrogen Content

The production of plant metabolites may be induced by chemical substances referred to as elicitors or by microbial bioeffectors. For instance, Algar *et al.* successfully applied bacterial strains to modify the biochemical profile and increase the level of isoflavones in soy [23]. Ion metals belong to elicitors and can initiate or enhance the synthesis of biologically active compounds in plants [24]. They disturb cellular homeostasis and lead to changes in the biochemical pathways, which in consequence modify accumulation of primary and secondary metabolites [25]. For example, a positive effect of fertilization with Mn and Fe on ginsenoside yield was observed [16]. In our present research, the influence of strontium ions on phytoestrogen production in soybeans was investigated. Initially, the extract from soybean seeds was analyzed to determine the phytoestrogen content. Daidzein and genistein were the main aglycones found after hydrolysis, with amounts of 575.1 ± 59.7 and 1016 ± 91.4 µg/g of dried plant material (70.84 ± 7.35, 125.2 ± 11.2 µg per seed), respectively. Then, the seeds were hydroponically cultivated with various concentrations of strontium. The Sr^2+^ content in the growth medium was established on the basis of our earlier research, and it was in the range from 0.5 to 3 mM [13]. After 21 days of cultivation, the plants were dried, extracted and hydrolyzed, and the amount of phytoestrogens was determined. The results obtained are presented in Table 1. Generally, the highest content of the investigated compounds was found in roots; however, formononetin was detected only in the aboveground part of the plants. Daidzein, genistein, and coumestrol constituted approx. 35.0%–42.3%; 35.7%–50.2% and 14.8%–23.7% of the total amount of phytoestrogens in roots, respectively; in turn, in shoots, daidzein and genistein were dominant and the other phytoestrogens were present only in a small amount (below 2.5%). Furthermore, for all strontium concentrations, a dose-dependent effect on phytoestrogen production in soybean plants was observed. The highest amount of phytoestrogens in shoots and roots was determined in soybeans treated with Sr^2+^ at a concentration of 2.0 mM. The level of daidzein, genistein and coumestrol in roots increased *ca.* 2.7, 4.1, and 2.0-fold, respectively, and in shoots, it increased *ca.* 4.2, 2.0, 3.9-fold, respectively, compared to the control (Table 2).

**Table 2:** The content of phytoestrogens (on gram of dried plant material ± SD) in different parts of *Glycine max* treated with various Sr^2+^ concentrations (*n* = 5). Formononetin was not detected in roots.

Sr^2+^ (mM)	Daidzein		Coumestrol		Genistein		Formononetin
	Roots (mg/g)	Shoots (µg/g)	Roots (mg/g)	Shoots (µg/g)	Roots (mg/g)	Shoots (µg/g)	Shoots (µg/g)
0 (control)	3.53 ± 0.231 ^e^	65.5 ± 5.67 ^d^	2.06 ± 0.154 ^d^	3.25 ± 0.52 ^d^	3.11 ± 0.304 ^d^	121.2 ± 16.9 ^b^	4.33 ± 0.53 ^c^
0.5	4.40 ± 0.250 ^d^	118.2 ± 8.15 ^c^	2.21 ± 0.201 ^c,d^	3.40 ± 0.57 ^c,d^	3.93 ± 0.363 ^c^	121.8 ± 17.0 ^b^	4.85 ± 0.60 ^c^
1.0	5.10 ± 0.361 ^c^	141.9 ± 12.04 ^b^	2.53 ± 0.209 ^b,c^	3.57 ± 0.58 ^c,d^	4.56 ± 0.426 ^b,c^	126.6 ± 17.1 ^b^	4.93 ± 0.61 ^c^
1.5	5.96 ± 0.386 ^b^	150.4 ± 13.45 ^b^	2.80 ± 0.267 ^b^	4.61 ± 0.57 ^c^	5.311 ± 0.460 ^b^	193.8 ± 24.3 ^a^	5.49 ± 0.73 ^c^
2.0	9.54 ± 0.694 ^a^	272.1 ± 19.99 ^a^	4.00 ± 0.314 ^a^	12.76 ± 1.38 ^b^	12.79 ± 1.02 ^a^	220.1 ± 30.3 ^a^	12.82 ± 1.30 ^a^
2.5	9.48 ± 0.819 ^a^	257.3 ± 20.67 ^a^	3.76 ± 0.415 ^a^	8.16 ± 0.93 ^a^	11.94 ± 0.96 ^a^	138.9 ± 20.1 ^b^	8.13 ± 0.97 ^b^
3.0	7.98 ± 1.80 ^a^	233.3 ± 46.33 ^a^	3.36 ± 0.788 ^a^	7.23 ± 1.39 ^a^	11.42 ± 2.53 ^a^	136.8 ± 26.1 ^b^	5.32 ± 1.09 ^c^

Different letters within each column indicate significant differences by Fisher’s test at *p* < 0.05.

A high positive correlation (*R* ≥ 0.82) between the content of isoflavones and the concentration of strontium in the medium in the range from 0 to 2.0 mM was observed (Table 3); however, at a higher Sr^2+^ concentration, the amount of isoflavones significantly decreased and a high negative correlation (*R* ≤ −0.89) was found. Moreover, a high variability of the results at a concentration of 3.0 mM was noted (RSD higher than 19%), which could be caused by the toxic effect of Sr^2+^ on plants.

**Table 3:** Correlation coefficient (r) between the concentration of strontium in medium and the content of phytoestrogens in *Glycine max* (*n* = 5).

Sr^2+^ Concentration Range (mM)	Daidzein		Genistein		Coumestrol		Formononetin
	Root	Shoot	Root	Shoot	Root	Shoot	Shoot
0–2.0	0.9815	0.9696	0.8996	0.8828	0.9784	0.8219	0.8352
2.0–3.0	−0.9981	−0.9991	−0.9400	−0.8992	−0.9754	−0.9421	−0.9873

**Figure 2:**
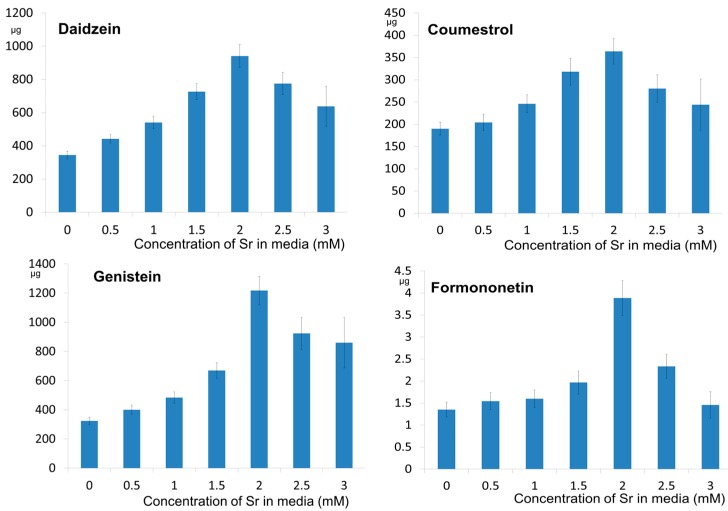
The effect of various strontium concentrations on the content (µg/plant) of daidzein, coumestrol, genistein and formononetin in *Glycine max* (*n* = 5).

The results of phytoestrogen quantification were recalculated and expressed as the average content in plant. The relationships between their amount and the concentration of strontium ions in the growth medium are presented in Figure 2.

After 21 days of cultivation, a significant increase in the amount of the investigated compounds in the plants was noted, compared with that in the seed. Moreover, at a strontium concentration of 2.0 mM, the content of phytoestrogens in the plants increased *ca.* 2.7, 1.92, 3.77, and 2.88-fold, for daidzein, coumestrol, genistein and formononetin, respectively, in comparison to the control.

To the best of our knowledge, the effect of strontium on the production of secondary plants metabolites has not been previously reported. Strontium is not a typical stress agent because, due to the physical and chemical similarity to calcium, it can partially replace Ca^2+^, which is an essential component for plant growth [26]. So far, the ability of plants to accumulate radioactive strontium isotope has been used for phytoremediation of contaminated soil [27,28]. On the other hand, strontium is useful for humans in therapy of osteoporosis [12]; thus, the accumulation thereof in plants may be beneficial in the context of the production of plant-based diet supplements enriched with strontium. Our study shows that strontium stress also has a positive impact on production of isoflavones in soybeans and may lead to obtaining plants with an increased level of phytoestrogens.

### 2.3. Cytotoxicity of Soybean Extract Obtained from Strontium-Stressed Plants

**Figure 3:**
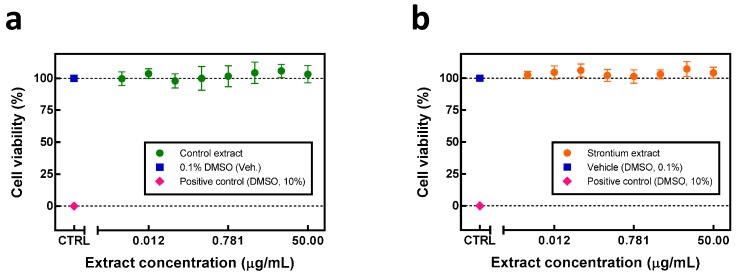
Dose-response effect of extracts from control and strontium-stimulated soybeans on viability of HepG2 liver cells assessed using tetrazolium reduction assay. The cells were treated with vehicle (DMSO, 0.1%) or with increasing concentrations of shoot extracts from (**a**) control and (**b**) 2 mM Sr^2+^-stressed soybeans. Ten percent solution of DMSO was used as a positive control for cytotoxicity. Viability of vehicle-treated cells was set at 100. Data show the average ± SD of three independent experiments, each performed in triplicate wells.

Tetrazolium dye reduction experiments were carried out to verify whether the extracts obtained from strontium-stressed soy were able to affect the growth and metabolic rates of cultured human cells *in vitro*. Human HepG2 cells were employed in this set of experiments as a model for hepatocytes, types of cells that are responsible for the first-pass metabolism and detoxification *in vivo*. No cytotoxic effects were observed in the cells after 24 h treatment with the extracts from the control soybean (Figure 3a). Similarly, extracts obtained from strontium-stressed plants were neutral to the growth rate of HepG2 cells (Figure 3b).

## 3. Materials and Methods

### 3.1. Materials and Reagents

All components of Hoagland’s medium, hydrochloric acid, and Sr(NO_3_)_2_ were from POCH (Gliwice, Poland). Daidzein, genistein, formononetin and coumestrol were purchased from Sigma-Aldrich (St. Louis, MO, USA). Methanol, HPLC-grade acetonitrile and trifluoroacetic acid (TFA) were purchased from Merck (Darmstadt, Germany). Water was deionized and purified by Ultrapure Milipore Direct-Q^®^ 3UV–R (Merck).

### 3.2. Hydroponic Cultivation of Plant Material

Soybeans seeds (*Glycine max* (L.) Merr.) were supplied by the Botanical Garden of Maria Curie-Skłodowska University in Lublin (voucher specimen No. 27-0705). The plants were germinated in a thermostat-controlled chamber for one week, and the seedlings were then transferred into a standard Hoagland’s nutrient solution. After 3 days, the growth medium was removed and replaced by Hoagland’s solution, modified with the addition of Sr^2+^ (the concentration of Sr^2+^ in the medium was 0-control, 0.5, 1.0, 1.5, 2.0, 2.5, and 3.0 mM). Twenty plants (5 plants in 4 repetitions) for each concentration of Sr^2+^ were grown for another 14 days at a day/night cycle of 16/8 h and 24/17 °C, respectively, with a relative humidity of 60% to 70% and a photosynthetic photon flux density of 150 µmol·m^−2^·s^−1^.

### 3.3. Sample and Standard Preparation

The plants were separated into roots and shoots, accurately weighed, dried and pulverized. 0.5 g of plant material was extracted three times with a fresh volume of methanol (3 × 20 mL) in an ultrasonic bath for 15 min. The combined extracts were concentrated to 10 mL, hydrolyzed, evaporated to dryness and dissolved in 10 mL or 100 mL of methanol for shoots and roots, respectively. The standard stock solutions at concentration 50 μg·mL^−1^ of daidzein, genistein, formononetin and coumestrol were prepared in methanol.

### 3.4. HPLC Condition

Chromatographic determination was performed on VWR Hitachi Chromaster 600 chromatograph (Merck) with a pump (5160), a degasser, a thermostat (5310), an autosampler (5260), a DAD detector (5430) and EZChrom Elite software. The extracts were analyzed on C18 reversed-phase column LiChrospher 100 (Merck) (25 cm × 4.0 mm i.d., 5 μm particle size), at a temperature of 30 °C. A 20 µL portion of the sample was injected. A mixture of acetonitrile (A) and water (B) with 0.025% of trifluoroacetic acid was used as a mobile phase. The gradient program was as follows: 0–10 min 20% A, 80% B; 10–30 min 25% A, 75% B; 30–60 min 35% A, 65% B. The flow rate of the eluent was 1.5 mL·min^−1^. The data were collected in a wavelength range from 200 to 400 nm. The chromatographic fractions eluted at a retention time characteristic for the investigated isoflavones were collected using a Foxy R1 fraction collector (Teledyne Isco, Lincoln, NE, USA) and analyzed by direct-injection mass spectrometry with electrospray ionization (micrOTOF-Q II, Bruker Daltonics, Bremen, Germany) using Compass DataAnalysis software Version 4.1.

### 3.5. Cell Culture

Human hepatocellular carcinoma HepG2 cells were purchased from American Type Culture Collection (Manassas, VA, USA). The cells were maintained in Minimum Essential Media with Earle’s salts, supplemented with 2 mM l-glutamine, 1 mM sodium pyruvate, 100 U/mL penicillin, 0.1 mg/mL streptomycin and 10% (*v*/*v*) fetal bovine serum (all from Thermo Fisher Scientific, Logan, UT, USA). The cells were cultured in a humidified atmosphere of 95% air and 5% CO_2_ at 37 °C. The medium was replenished every 3 days, and cells were subcultured after reaching 70%–80% confluence.

### 3.6. Cell Viability Assessment

Cytotoxic effects of leaf and root extracts obtained from strontium-stressed soybeans were studied using a colorimetric CellTiter 96 AQueous One Solution Cell Proliferation Assay (Promega, Mannheim, Germany). HepG2 cells were seeded in 96-well plates at 2 × 104 cells per well at cultured for 24 h. Then, the cells were treated with increasing concentrations of the extracts in a serum-free medium or with a vehicle (DMSO, 0.1%) as a control. A 10% solution of DMSO was used as a positive control for cytotoxicity. After 24 h of incubation, CellTiter reagent (20 µL/well) was added. Two hours later, the absorbance was recorded at 490 nm using an ELx800 microplate reader (BioTek Instruments, Winooski, VT, USA). Three independent experiments were carried out in triplicates.

### 3.7. Statistical Analysis

Data were analyzed using STATISTICA ver.10 (StatSoft, Inc., Tulsa, OK, USA). Differences between the means across treatment groups were evaluated using a one-way analysis of variance (ANOVA) followed by Fisher’s LSD test. Data with *p*-value less than 0.05 were considered statistically significant.

## 4. Conclusions

Our results showed that strontium ions act as an elicitor and stimulate the production of isoflavones and coumestrol in *G. max*. The amount of phytoestrogens in soybean plants strongly depended on the concentration of strontium ions in the growth medium. Their concentrations in both shoots and roots increased significantly with the increasing content of strontium ions and reached the maximal level at 2.0 mM. In conclusion, our studies show that strontium ion stress has a positive impact on the production of phytoestrogens in soybeans and may generate plants that have increased levels of phytoestrogens and that are simultaneously enriched with strontium ions.
